# Cadmium-Induced Physiological Responses, Biosorption and Bioaccumulation in *Scenedesmus obliquus*

**DOI:** 10.3390/toxics12040262

**Published:** 2024-03-31

**Authors:** Pingping Xu, Xiaojie Tu, Zhengda An, Wujuan Mi, Dong Wan, Yonghong Bi, Gaofei Song

**Affiliations:** 1 State Key Laboratory of Freshwater Ecology and Biotechnology, Institute of Hydrobiology, Chinese Academy of Sciences, Wuhan 430072, China; xupingping@ihb.ac.cn (P.X.); miwj@ihb.ac.cn (W.M.); wandong@inb.ac.cn (D.W.); biyh@ihb.ac.cn (Y.B.); 2 University of Chinese Academy of Sciences, Beijing 100049, China; 3 Geophysical Exploration Brigade of Hubei Geological Bureau, Wuhan 430056, China; tuxj@ihb.ac.cn; 4 College of Life Science, Wuhan University, Wuhan 430072, China; 2020300002057@whu.edu.cn

**Keywords:** cadmium, *Scenedesmus obliquus*, response, biosorption, bioaccumulation

## Abstract

Cadmium ion (Cd^2+^) is a highly toxic metal in water, even at low concentrations. Microalgae are a promising material for heavy metal remediation. The present study investigated the effects of Cd^2+^ on growth, photosynthesis, antioxidant enzyme activities, cell morphology, and Cd^2+^ adsorption and accumulation capacity of the freshwater green alga *Scenedesmus obliquus*. Experiments were conducted by exposing *S. obliquus* to varying concentrations of Cd^2+^ for 96 h, assessing its tolerance and removal capacity towards Cd^2+^. The results showed that higher concentrations of Cd^2+^ (>0.5 mg L^−1^) reduced pigment content, inhibited algal growth and electron transfer in photosynthesis, and led to morphological changes such as mitochondrial disappearance and chloroplast deformation. In this process, *S. obliquus* counteracted Cd^2+^ toxicity by enhancing antioxidant enzyme activities, accumulating starch and high-density granules, and secreting extracellular polymeric substances. When the initial Cd^2+^ concentration was less than or equal to 0.5 mg L^−1^, *S. obliquus* was able to efficiently remove over 95% of Cd^2+^ from the environment through biosorption and bioaccumulation. However, when the initial Cd^2+^ concentration exceeded 0.5 mg L^−1^, the removal efficiency decreased slightly to about 70%, with biosorption accounting for more than 60% of this process, emerging as the predominant mechanism for Cd^2+^ removal. Fourier transform infrared correlation spectroscopy analysis indicated that the carboxyl and amino groups of the cell wall were the key factors in removing Cd^2+^. In conclusion, *S. obliquus* has considerable potential for the remediation of aquatic environments with Cd^2+^, providing algal resources for developing new microalgae-based bioremediation techniques for heavy metals.

## 1. Introduction

Cadmium (Cd), a non-essential trace metal element, is a major toxic metal pollutant even at low concentrations [1,2]. Cd can enter natural water through various sources, causing physiological and morphological changes in animals and plants, especially in microalgae, which are primary producers of water ecosystems [3]. Cadmium has been shown to affect growth [4], reproduction, physiological and biochemical processes in microalgae [5]. For example, cadmium affects photosynthesis, leads to chloroplasts and mitochondria damage, upregulates antioxidant capacity, and alters cellular ultrastructure [6,7]. However, the same heavy metal has different effects on algal cell growth and metabolism.

Despite the toxicity of Cd, microalgae have evolved complex resistance mechanisms. These mechanisms include extracellular immobilization, intracellular sequestration, and detoxification of metal ions [8]. The cell wall and extracellular polymeric substances (EPS) can adsorb toxic metals through electrostatic interactions, complexation, reduction, ion exchange, and surface precipitation [9,10]. In the cytoplasm, amino acids, chelating peptides, and organic acids are capable of chelating heavy metals [11]. Furthermore, cells can regulate reactive oxygen species (ROS) induced oxidative damage through the antioxidant system [5]. Different algae may use different strategies to counteract metal toxicity due to differences in living environment, structure, and physiological level.

The Genus *Scenedesmus* is a group of green algae widely distributed in aquatic ecosystems with the ability to accumulate high concentrations of metal ions. Some studies have investigated the interaction of *Scenedesmus *sp. with Cd, focusing on heavy metal risk assessment, biotechnology of heavy metal removal by microalgae, and integration of microalgae biofuel with wastewater treatment [12]. Other studies have investigated the response mechanisms of *Scenedesmus obliquus* to Cd, for example, Mangal and Nguyen et al. [13] used an untargeted metabolomics approach to characterize the response of algal metabolites to cadmium stress. Previously, we studied the interaction between algal organic matter (AOM) and Cd^2+^ in *S. obliquus* and elucidated the detoxification mechanism of AOM [14]. Innovations in biotechnology have led to new methods for heavy metal removal, such as the immobilization of microalgae cells for the treatment of cadmium-rich wastewater [15] and the development of fixed-bed biosorption systems for Cd^2+^ removal [16]. In addition, the combination of microalgal biomass production with wastewater treatment [17] has been proposed as a cost-effective bioremediation strategy.

Despite numerous studies on Cd toxicity and adsorption by *Scenedesmus *sp., a comprehensive analysis incorporating various effects and mechanisms has been lacking. Therefore, we conducted a 96-h toxicological experiment with *S. obliquus* under varying Cd^2+^ concentrations to investigate physiological changes such as morphology, structure, optical density, pigment content, and antioxidant enzyme activities under Cd stress, including the identification of Cd damage sites through chlorophyll fluorescence. We also measured Cd^2+^ concentrations on the cell surface, inside the cell, and in the medium to assess biosorption and bioaccumulation capacities. In addition, extracellular polymeric substances were quantitatively and qualitatively analyzed to explore their mechanisms of action in biosorption. The physiological parameters measured in this study can be used for environmental risk assessment, which will help to develop more effective guidelines for environmental protection and provide algal resources and theoretical references for the future development of heavy metal removal technologies using *S. obliquus*.

## 2. Materials and Methods

### 2.1. The Algal Cultivation

*Scenedesmus obliquus* (F-417) was purchased from Freshwater Algae Culture Collection at the Institute of Hydrobiology (FACHB), Chinese Academy of Sciences (Wuhan, China). Algal cells were cultivated in BG11 medium [18] using aseptic techniques in 250 mL Erlenmeyer flasks and placed in a PGX-350B (Yanghui Co., Ltd., Ningbo, China) light incubator with a light intensity of 30 μmol photons m^−2^ s^−1^ (white fluorescent lamp) at 25 ± 1 °C, following a day/night cycle of 12:12 h.

### 2.2. Cd Toxicity Test

Considering prior findings the metal ion chelator EDTA (ethylenediaminetetraacetic acid, at 1 mg L^−1^) mitigates cadmium toxicity without significantly affecting algal growth and photosynthesis [19]. We opted for a modified BG11 medium devoid of EDTA for our experiments. A 1000 mg L^−1^ Cd^2+^ stock solution was prepared by dissolving CdCl_2_ in sterile water, which was then sterilized and filtered through a 0.22 μm filter for purity.

Algal cells, in their logarithmic growth phase, were harvested and resuspended in 250 mL Erlenmeyer flasks filled with 150 mL of the sterilized BG11 medium. The initial optical density (OD_680_ nm) was set to 0.3, corresponding to a cell density of approximately 3 × 10^6^ cells mL^−1^. To simulate varying environmental cadmium concentrations, cadmium stock solutions were added to achieve final Cd^2+^ concentrations of 0.005, 0.01, 0.05, 0.5, 5, and 10 mg L^−1^ in the culture medium, referencing heavy metal wastewater discharge standards and existing research on cadmium’s effects on algae. A control group was maintained in a Cd^2+^-free BG11 medium. Each treatment, including the control, was replicated three times to ensure reliability, and the cultures were incubated for 96 h under standard conditions, mirroring the control setup.

### 2.3. Algal Growth

To determine the effect of Cd^2+^ on the growth of *S. obliquus*, the growth parameters such as optical density (OD), chlorophyll *a* (Chl *a*), and chlorophyll *b* (Chl *b*) were determined for each group of flasks. The optical density at 680 nm was measured using a spectrophotometer (UV-1780, Shimadzu, Kyoto, Japan) at 0, 24, 48, 72 and 96 h.

The concentrations of Chl *a* and Chl *b* were determined according to Fan [20]. *S. obliquus* cells were harvested by centrifugation (12,000× *g* for 3 min) at 0, 24, 48, 72 and 96 h. Cells were then suspended in 1 mL of 100% methanol and homogenized with a cryogenic grinder at 4 °C for 10 min (steps of 30 s) and 70 Hz which using an automatic sample cryogenic grinder (Shanghai Jing Xin, Shanghai, China). The extracted material was kept in a water bath at 45 °C in the dark for 30 min and then centrifuged at 10,000× *g* for 3 min to remove cell debris before measuring the absorbance of the supernatants at 470, 646, and 663 nm with a UV-Vis spectrophotometer (UV-1780, Shimadzu, Japan), using 100% methanol was used as blank reference.

The toxic responses of test microalgae to cadmium were analyzed by the EC_50_ values, which are the doses causing 50% inhibition of microalgae. The EC_50_ value after 96 h exposure was calculated by fitting the DoseResp curve based on pigment contents (Equation (1)) using Origin 2023 (OriginLab Corporation, Northampton, MA, USA) according to Li et al. [21].

(1) $$y=A_{1}+(A_{2}-A_{1})/({1+10}^{\left(\log_{X}0-X\right)}P)$$

where: A_1_ is bottom asymptote, A_2_ is top asymptote, Log_X_0: Center, and P is Hill slope, and EC_50_ = 10^LOGx0^.

### 2.4. Photosynthesis and Respiration

The rate of photosynthetic oxygen evolution and respiratory oxygen consumption of algal cells at 96 h was determined using a liquid-phase oxygen measurement system (Chlorolab 2, Hansatech Ltd., King’s Lynn, UK). The temperature of the assay was 25 °C, and the respiratory oxygen consumption rate of the cells was determined preferentially under dark conditions, followed by turning on the light source and determining the rate of photosynthetic oxygen evolution under 400 μmol photons m^−2^ s^−1^ light (saturated light intensity) [22]. A smooth curve was chosen to calculate the apparent photosynthetic oxygen evolution rate and the respiratory oxygen consumption rate.

### 2.5. Chlorophyll Fluorescence

To access the effect of Cd^2+^ on photosynthesis in *S. obliquus*, the kinetics of chlorophyll fluorescence induction after 96 h of incubation in different concentrations of Cd^2+^ were measured using an Aqua-Pen-100 fluorometer (Photo Systems Instruments, Brno, Czech Republic) according to Zhang [23]. Briefly, a 3 mL sample of algal solution (OD_680_ nm = 0.5) was dark-adapted for 15 min, and then the minimum fluorescence level (Fo) was first measured under weak red light (<1 μmol m^−2^ s^−1^) irradiation at 450 nm. The maximum fluorescence level (Fm) was then measured using saturating flash irradiation at 400 μmol m^−2^ s^−1^. A typical kinetic curve for the induction of fast chlorophyll fluorescence has four phases: O, J, I, and P, with the J, I, and P phases occurring at approximately 2, 30, and 400 ms, respectively. When the PS II action centers are fully opened, the fluorescence of all electron acceptors (Q_A_, Q_B_, PQ, etc.) in the maximally oxidized state is called the initial fluorescence and is defined as the point O. The fluorescence intensity in the J stage represents the accumulation of Q_A_^−^Q_B_ and Q_A_^−^Q_B_^−^, and the intermediate I stage reflects the accumulation of Q_A_^−^Q_B_^2−^. Point P represents the fluorescence of PS II electron acceptors in the maximally reduced state. For comparison, we normalized the fluorescence signals of different samples to relative variable fluorescence (Vt) using the mathematical formula Vt = (Ft − Fo)/(Fm − Fo) and analyzed using the JIP–test [24]. The relevant parameters are presented in Table 1.

### 2.6. Q_A_^−^ Reoxidation Kinetics

Q_A_^−^ reoxidation measurement can reflect the inhibition of electron transport on the receptor side of PSII. The positions of the potential gradients for the “donor side of PSII”, “acceptor side of PSII”, “Q_B_ binding site”, and “reduction of plastoquinone” are shown in the schematic diagram of the electron transport chain in Figure S1 [24] of Supplementary Material. To further localize the site of action of Cd^2+^ on the photosystem of *S. obliquus*, the kinetics of Q_A_^−^ reoxidation was determined by taking 3 mL of algal solution (OD_680_ nm = 0.5) and dark-adapted for 15 min using FL–6000 dual–modulation kinetic fluorometer (Photon Systems Instruments, Brno, Czech Republic). The fitting of Q_A_^−^ reoxidation kinetics curves is referred to in Beauchemin’s paper [25].

Q_A_^−^ reoxidation kinetic curves were fitted by the following Equation (2):

(2) $$F\left(t\right)=A_{1}\mathrm{ex}p\left(-t/T_{1}\right)\;+A_{2}\exp\left(-t/T_{2}\right)\;+A_{3}\exp\left(-t/T_{3}\right)+A_{0}$$

where F(t) is the variable fluorescence yield, A_0_ to A_3_ are the amplitudes, and T_1_ to T_3_ are the time constants from which the half-life values can be calculated as t_1/2_ = ln 2T.

After fitting, the fast, middle, and slow phases of the fluorescence decay process of Q_A_^−^ reoxidation were obtained; corresponding to the amplitudes (A_1_, A_2_, A_3_) and time constants (T_1_, T_2_, T_3_), respectively. The half−life values t_1/2_ = ln 2T for the fast, middle, and slow phases can be calculated from the time constants T_1_ to T_3_. The fast phase is the transfer of electrons from Q_A_^−^ to Q_B_, that is, Q_A_ transfers electrons to Q_B_^−^/Q_B_, when the Q_B_ site in the reaction center has bound PQ molecules (oxidized or semi-reduced state) before the flash, with a half−life of a few hundred microseconds. The mid−phase is also the transfer of electrons from Q_A_^−^ to Q_B_, but the re-oxidation of Q_A_^−^ is limited by the rate of diffusion of the PQ molecules into the empty Q_B_ site, and the process in the mid−phase usually lasts for a few milliseconds, and the slow phase arises from the reverse charge recombination of the Q_A_^−^ with the OEC S2 state, and usually lasts for a few seconds to several tens of seconds.

### 2.7. Total Protein Quantification

Total proteins were extracted using the Plant Protein Extraction Kit (Cowin, Beijing, China) [26]. Briefly, 1 mL of algal solution was centrifuged at 4000× *g* for 5 min. The cells were then suspended in 1 mL of plant protein extraction reagent and homogenized with a cryogenic grinder at 4 °C for 10 min (steps of the 30 s) and 70 Hz (Shanghai Jing Xin, China). The extracted material was kept in an ice bath for 30 min and then centrifuged at 13,400× *g* for 20 min to remove cell debris and protein content was determined using the BCA protein quantification kit (Cowin, Beijing, China), according to the method of Rogatsky [27].

### 2.8. Reactive Oxygen Species Measurement

Reactive oxygen species (ROS) were determined according to Aranda et al. [28]. The ROS content was determined using the Reactive Oxygen Species Assay Kit (Beyotime Biotechnology, Shanghai, China) according to the manufacturer’s instructions. 1 mL of cells was collected using centrifugation at 3500× *g* for 5 min; the cell paste was resuspended in 1 mL of PBS plus 10 μM 2′,7′-dichlorofluorescein-diacetate (DCFH-DA) and cultivated in the dark for 30 min at 37 °C. After incubation, the cells were centrifuged at 3500× *g* for 5 min and washed twice with PBS. 2′,7′-dichlorofluorescein (DCF) fluorescence (485/525 nm) was determined using a Multi-Mode Microplate reader (VICTOR Nivo; PerkinElmer, Turku, Finland) and normalized to chlorophyll fluorescence (430/670 nm).

### 2.9. Determination of Antioxidant Enzyme Activities

The superoxide dismutase (SOD) and catalase (CAT) were measured using detection kits (Jiancheng Institute of Biological Engineering, Nanjing, China). A 5 mL culture sample was taken from each experimental group and centrifuged at 3500× *g* for 10 min. The cells were suspended in 5 mL of a 10 mM phosphate-buffered saline (PBS) solution (pH 7.0). Then homogenized with a cryogenic grinder at 4 °C for 10 min (steps of the 30 s) and 70 Hz, followed by another 10 min of centrifugation (3500× *g*). The supernatant, serving as the enzyme extract, was analyzed for SOD and CAT activities. SOD activity was determined using the WST-1 method, in which WST-1 reacts with the superoxide anion catalyzed by xanthine oxidase to form the water-soluble methazazanine dye, a reaction step that can be inhibited by SOD, and the enzyme activity of SOD was calculated by colorimetric analysis of the WST-1 product at 450 nm, expressed as U mg^−1^ protein. CAT activity was quantified spectrophotometrically by reacting hydrogen peroxide with ammonium molybdate to form a yellow complex, and the enzyme activity of CAT was calculated by colorimetric analysis of the product at a wavelength of 405 nm. CAT activity is defined as the amount of enzyme that catalyzes the conversion of 1 micromole of H_2_O_2_ per minute per milligram of protein as 1 U.

### 2.10. Cellular Morphology

To further understand the response of *S. obliquus* to Cd^2+^ stress, the morphology of microalgal cells was observed by scanning electron microscopy (SEM, Hitachi S-4800, Hitachi, Ltd., Tokyo, Japan) and transmission electron microscopy (TEM, Hitachi HT-770, Ltd. Tokyo, Japan). Microalgae cells were fixed with 2.5% glutaraldehyde at 4 °C for 24 h, then washed with phosphate buffer (0.05 M, pH 7.0), dehydrated with 30%, 50%, 70%, 90%, and 100% ethanol, and finally sputtered with gold before SEM analysis. The morphology of the cells was observed by SEM.

For TEM analysis, cell samples from control and Cd-treated cultures respectively were centrifuged, fixed, dehydrated, sectioned, and stained. The ultrastructural changes of *S. obliquus* cells were observed by TEM.

### 2.11. Fourier Transform Infrared Spectroscopy

To analyze the interactions between Cd^2+^ and the functional groups present in the cell walls of microalgae, a 50 mL sample of thoroughly mixed algal solution was collected at 96 h. This sample was then freeze-dried and ground into a fine powder, which was subsequently mixed evenly with potassium bromide at a 1:100 ratio. The mixture was then compressed into tablets, which were utilized for Fourier Transform Infrared Spectroscopy analysis using a Perkin Elmer Frontier instrument (Perkin Elmer Frontier, Perkin Elmer Inc., Waltham, MA, USA).

### 2.12. Extraction and Compositional Analysis of EPS

EPS secreted by microalgal cells provides binding sites for heavy metals, thereby increasing the adsorption capacity of heavy metals in aquatic environments. To explore whether cadmium stress induces the secretion of EPS in algae, the supernatant separated from the algal suspension by centrifugation (10,000× *g* for 15 min) was the EPS, which was then filtered through a 0.22 μm acetate cellulose membrane to remove microalgal cells and other residues [29]. The protein and polysaccharide contents of EPS were determined using the BCA assay [26] and the phenol sulfuric acid digestion [30] method, respectively.

### 2.13. Cadmium Distribution

Cd^2+^ added to the flasks were distributed on the cell surface, inside the cells, and in the medium, with the sum of the percentages of Cd^2+^ in the three fractions being 100%. To determine the Cd^2+^ distribution and proportions, 1 mL of algal solution was collected at 0, 24, 48, 72, and 96 h. The solution was centrifuged for 3 min at 10,000× *g*. The cell-free supernatant was filtered through a 0.22 μm Millipore filter and the concentration of Cd^2+^ in the medium was measured by ICP-MS (Agilent 8900, Tokyo, Japan). The cell pellet was washed with 2 mM EDTA for 10 min and centrifuged at 10,000× *g* for 3 min to remove Cd^2+^ adsorbed on the biomass surface. The EDTA-washed cell pellet was initially digested in 2 mL of concentrated HNO_3_ at 100 °C for 1 h and further digested at 150 °C before the samples were cooled to room temperature. The digested samples were diluted and filtered appropriately and measured intracellular Cd^2+^ concentration by ICP-MS (Agilent 8900, Tokyo, Japan). Cadmium adsorbed on the cell surface was determined by the difference between the initial and final cadmium concentrations in the culture medium.

Cd accumulation percentage was determined by Equation (3):

(3) $$\mathrm{Cd}\;\mathrm{accumulation}\;\left(\%\right)=C_{\alpha }/{\;C}_{i\;}\;\times \;100$$

where C_α_ is the intracellular Cd^2+^ concentration (mg L^−1^) and C_i_ is the initial concentration (mg L^−1^) of Cd^2+^ in the culture medium.

Cd adsorption percentage was determined by Equation (4):

(4) $$\mathrm{Cd}\;\mathrm{adsorption}\;\left(\%\right)={(C}_{i}-C_{f}-C_{\alpha })/C_{i}\;\times \;100$$

where C_α_ is the intracellular Cd^2+^ concentration (mg L^−1^) and C_i_ and C_f_ are the initial and final concentrations (mg L^−1^) of Cd^2+^ in the culture medium, respectively.

Cadmium in the medium percentage was determined by Equation (5):

(5) $$\mathrm{Cd}\;\mathrm{in}\;\mathrm{the}\;\mathrm{medium}\;\left(\%\right)=C_{f}/{\;C}_{i}\;\times \;100$$

where C_i_ and C_f_ are the initial and final concentrations (mg L^−1^) of Cd^2+^ in the culture medium, respectively.

### 2.14. Statistical Analysis

Statistical analyses were conducted using Origin 2022 (OriginLab Corporation, Northampton, MA, USA) and SPSS 22.0 (IBM SPSS, Chicago, IL, USA) software. Significant differences between the control and treated cultures were analyzed using a one-way ANOVA test followed by the LSD test or the Games-Howell test. Values were considered significant at *p* < 0.05.

## 3. Results

### 3.1. Cellular Morphology

**Figure 1:**
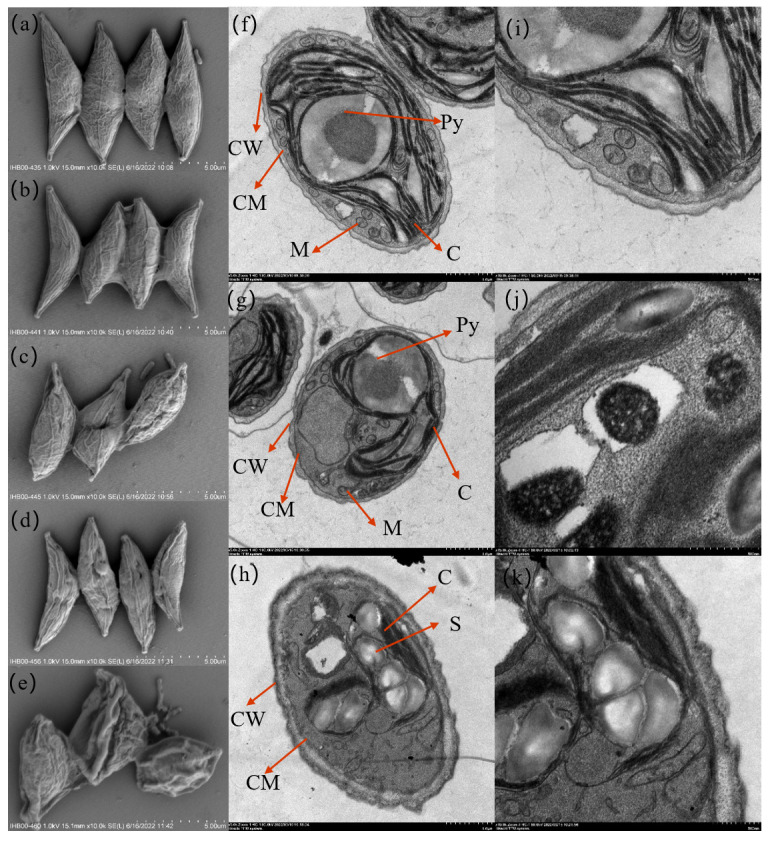
Morphology of *S. obliquus* treated with different concentrations of Cd^2+^: (**a**) SEM images with control, (**b**) SEM images of 0.05 mg L^−1^ Cd^2+^ treatment for 48 h; (**c**) SEM images of 10 mg L^−1^ Cd^2+^ treatment for 48 h; (**d**) SEM images of 0.05 mg L^−1^ Cd^2+^ treatment for 96 h; (**e**) SEM images of 10 mg L^−1^ Cd^2+^ treated 96 h; (**f**,**i**) TEM images with control; (**g**,**j**): TEM images of 0.05 mg L^−1^ Cd^2+^ treated 96 h; (**h**,**k**) TEM images of 10 mg L^−1^ Cd^2+^ treated 96 h. The alphabets in the image depict CW: cell wall, CM: cell membrane, C: chloroplast, M: mitochondrion, Py: Pyrenoid, and S: starch granules.

Scanning electron microscopy (SEM) and transmission electron microscopy (TEM) showed significant changes in the morphological structure of *S. obliquus* before and after Cd stress. The SEM images in Figure 1a depicted the control group of *S. obliquus*. SEM images showed that cells of *S. obliquus* were spindle-shaped, being broadest near the middle and tapering toward both ends. These nonmotile cells typically formed coenobia, arranged in rows of 2 or 4 cells. The individual cells were approximately 8–10 μm long and 3–5 μm wide. The TEM image of the control group was shown in Figure 1f,i. Ultrathin sections of untreated cells displayed typical organelles, structures, and inclusions characteristic of this species. The cell wall was thin, smooth, and wavy, with a large protein nucleus surrounded by starch ridges visible. The chloroplast occupied most of the cell volume, with numerous thylakoid membranes parallel to each other, occasionally containing starch granules in the thylakoid space. Many small mitochondria and few vacuoles were observed in the cytoplasm.

Under Cd^2+^ stress, most of the coenobium gradually became unicellular. The cells shriveled with surface folds deepened (Figure 1b,c,d,e). At high Cd^2+^ concentration (10 mg L^−1^), cells lysed and died, and the intracellular materials were released. After 96 h Cd exposure, TEM showed thickened cells, plasmolysis, chloroplast shrinkage, photosynthetic lamella adherence, mitochondrial deformation, or even disappearance (Figure 1g,h,j,k). Furthermore, the starch granules increased in number and volume, with some cells lysing and releasing their contents. In addition, the non-membranous electron-dense inclusions were observed in the vacuoles.

### 3.2. Growth and Tolerance

**Figure 2:**
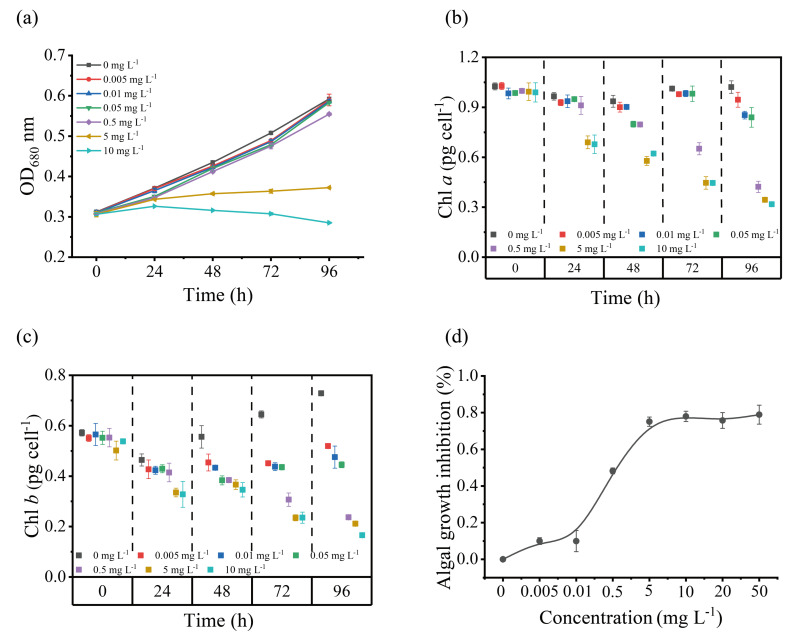
Effects of exposure to different concentrations of Cd^2+^ for 96 h on the growth and pigments of *S. obliquus*: (**a**) growth curves of *S. obliquus* on media with different Cd^2+^ concentrations, (**b**) Chl *a* content after 0, 24, 48, 72, 96 h incubation, (**c**) Chl *b* content after 0, 24, 48, 72, 96 h incubation, (**d**) The dose–effect curve for 96 h after exposure to different concentrations of Cd^2+^ in *S. obliquus*. Each data point is mean ± SE of three replicates.

To investigate the tolerance of *S. obliquus* to different Cd^2+^ concentrations, the growth performance under Cd^2+^ stress was studied. It was found that when the Cd concentration was lower than EC_50_, there was no significant effect, and when it was higher than EC_50_, the optical density and pigment were significantly affected. As shown in Figure 2a, the growth of *S. obliquus* cells maintained a continuously increasing trend under the low Cd^2+^ concentration (< 0.05 mg L^−1^), and while at high Cd^2+^ concentrations (5 and 10 mg L^−1^), the growth of *S. obliquus* cells was significantly inhibited (*p* < 0.05). As shown in Figure 2b,c, compared to the control group, there was no significant difference in Chlorophyll *a* (Chl *a*) at Cd concentrations lower than 0.5 mg L^−1^; however, the inhibitory effects were significant when the Cd^2+^ concentration was higher than 0.5 mg L^−1^ during 72 h. After 96 h of stress, Chl *a* was significantly decreased in all Cd-treated groups (*p* < 0.05). Chlorophyll *b* (Chl *b*) also decreased significantly from 48 h of Cd stress treatment (*p* < 0.05). EC_50_ value was determined by measuring the growth of the algae at various Cd^2+^ concentrations (0–50 mg L^−1^). As shown in Figure 1d, the EC_50_ value was 0.41 ± 0.03 mg L^−1^ at 96 h.

### 3.3. Photosynthesis and Respiration

**Figure 3:**
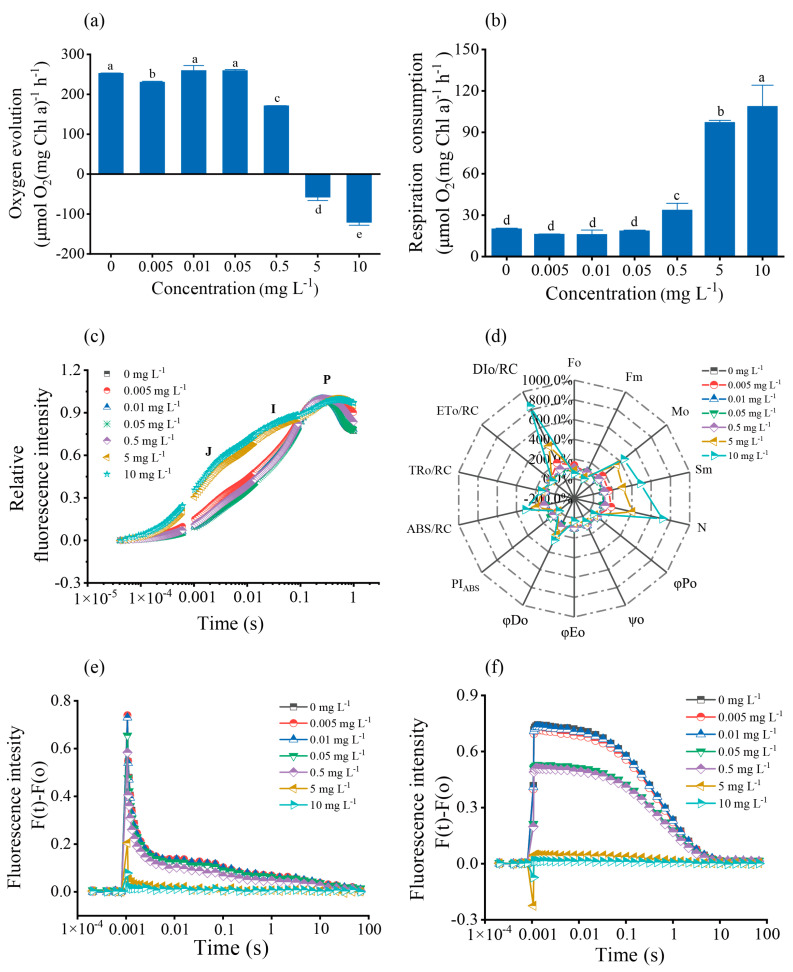
Effects of exposure to different concentrations of Cd at 96 h on photosynthesis and respiration of *S. obliquus*: (**a**) oxygen evolution rate, (**b**) respiration consumption rate, (**c**) relative variable fluorescence curve, (**d**) JIP test parameters, (**e**) Q_A_^−^ reoxidation kinetic curves: fluorescence decay in the absence of DCMU, (**f**) fluorescence decay in the presence of 20 μM DCMU. Each data point is mean ± SE of three replicates. Different letters above columns represent statistically significant differences, *p* < 0.05.

Photosynthesis and respiration are pivotal metabolic activities of photosynthetic organisms, so we explored the effects of cadmium stress on these metabolic activities and found that high levels of cadmium significantly impair both photosynthesis and respiration in algae. These includes a notable reduction in the photosynthetic oxygen evolution rate and disruption of electron transfer during photosynthesis. As shown in Figure 3a, there was a significant inhibition of photosynthetic oxygen evolution of *S. obliquus* observed with an increasing Cd^2+^ concentration in the medium (*p* < 0.05), particularly at Cd^2+^ concentrations of 0.5, 5, and 10 mg L^−1^ compared to the control. Simultaneously, the rate of respiratory oxygen consumption exhibited an upward trend with increasing cadmium concentrations (Figure 3b).

The effect of Cd on photosynthesis was further elucidated through rapid chlorophyll fluorescence−induced kinetic analysis. Before Cd^2+^ treatment, *S. obliquus* showed a typical fast chlorophyll fluorescence induction kinetic curve with four phases: O, J, I, and P. However, under high Cd^2+^ stress (5 and 10 mg L^−1^), as shown in Figure 3c and Figure S2, there was a pronounced increase in the relative variable intensity at the J step (V_J_) was enhanced, indicating that electron transfer from Q_A_^−^ to Q_B_ was inhibited. In addition, the emergence of a new phase (K) signaled a disruption in the oxygen-evolving complexes (OEC) on the electron donor side, highlighting the detrimental effects of high cadmium stress on the photosynthetic apparatus.

To further investigate the effect of cadmium stress on electron transfer on the PSII receptor side of algal cells, we performed a JIP test analysis. As shown in Table S1 and Figure 3d, both the minimum fluorescence intensity (Fo), the maximum fluorescence intensity (Fm), the maximum quantum yield for primary photochemistry (φ_Po_), the probability that a trapped exciton moves an electron into the electron transport chain beyond Q_A_^−^ (ψ_o_), the quantum yield for electron transport (φ_Eo_), the electron transport flux per reaction center (ETo/RC), and the performance index on absorption basis (PI_ABS_) showed a stepwise decrease between 5 and 10 mg L^−1^ of culture. Conversely, parameters such as the number of times Q_A_ was restored from the time (N), the quantum yield for dissipated energy (φ_Do_), the dissipated energy flux per reaction center (DIo/RC), the absorption flux per reaction center (ABS/RC), the trapped energy flux per reaction center (TRo/RC), and the approximated initial slope of the fluorescence transient (Mo) increased significantly. These findings suggest that Cd^2+^ concentrations up to 0.5 mg L^−1^ do not significantly affect photosynthetic activity, whereas higher concentrations may decrease the electron transfer efficiency around PSII and inhibit the electron transfer from Q_A_^−^ to Q_B_.

To corroborate these observations, Q_A_^−^ reoxidation kinetics were analyzed. As shown in Figure 3e,f, the time constant of the fast phase extended from 22.4 μs in control cells to 27.87 μs in cells exposed to 0.05 mg L^−1^ of Cd^2+^ (Table S2), indicating that the concentration of Cd^2+^ up to 0.5 mg L^−1^ did not significantly affect photosynthetic activity, higher concentrations adversely affected the electron transfer from Q_A_^−^ to Q_B_.

### 3.4. Antioxidant Enzymes

**Figure 4:**
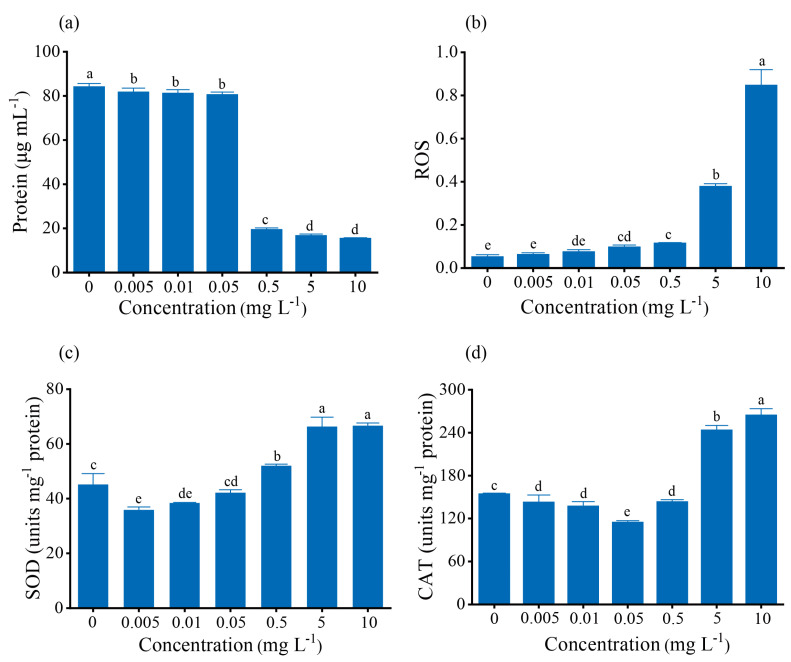
Effects of exposure to different concentrations of Cd^2+^ at 96 h on the antioxidant system of *S. obliquus*: (**a**) protein content, (**b**) ROS, (**c**) superoxide dismutase (SOD) activity, and (**d**) catalase (CAT) activity. Each data point is mean ± SE of three replicates. Different letters above columns represent statistically significant differences, *p* < 0.05.

To elucidate the survival mechanism adopted by the cells under Cd^2+^ toxicity, we investigated the modulation of protein, reactive oxygen species (ROS), catalase (CAT), and superoxide dismutase (SOD) (Figure 4). It was found that Cd stress induced oxidative stress in algae, prompting the activation of antioxidant defense mechanisms to counteract Cd stress. Notably, the total protein content in algae decreased significantly with the increase of Cd^2+^ concentration after 96 h of exposure (Figure 4a). In contrast, a significant increase in ROS was observed when concentrations of Cd^2+^ ≥ 0.05 mg L^−1^ were present (Figure 4b). Interestingly, the activities of both SOD and CAT first decreased and then increased (Figure 4b,c). Specifically, SOD activity decreased to a minimum at a Cd^2+^ concentration of 0.005 mg L^−1^, while CAT activity reached its minimum at a Cd^2+^ concentration of 0.05 mg L^−1^, with both enzymes showing increased activity at 5 and 10 mg L^−1^.

### 3.5. Biosorption and Accumulation

**Figure 5:**
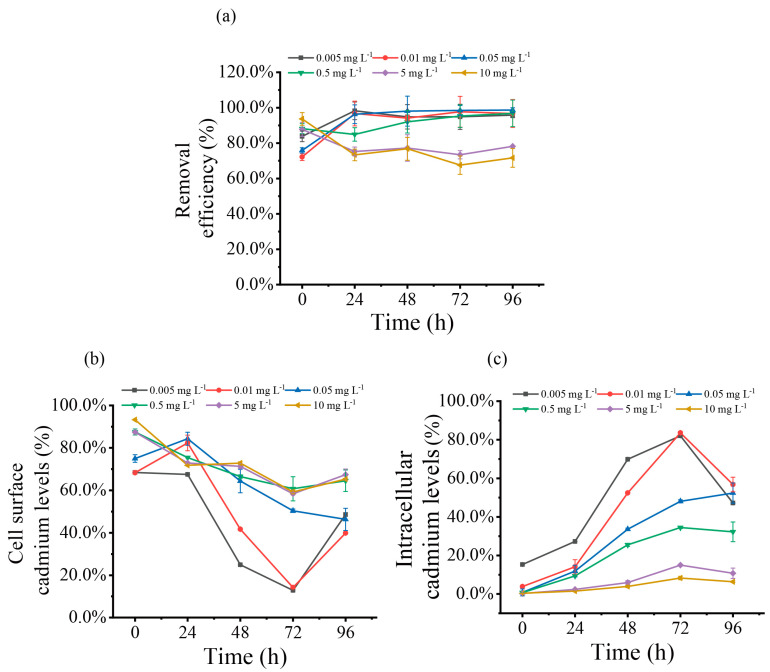
Effect of exposure to different concentrations of Cd^2+^ on the distribution of cadmium on the cell surface, intracellular, and in the medium of *S. obliquus*: (**a**) total Cd levels removed by cells over time; (**b**) Cd levels adsorbed on the cell surface over time; and (**c**) Cd levels accumulated intracellularly over time. Each data point is the mean ± SE of three replicates.

To elucidate the distribution of cadmium and its ability to remove cadmium, the concentration of Cd^2+^ on the cell surface, intracellularly, and in the culture medium at different times was determined during the time course experiment described in Figure 2a. The results showed that the adsorption, accumulation, and removal percentage of cadmium by *S. obliquus* varied depending on the cadmium concentration and duration of exposure. Figure 5a showed the cadmium removal efficiency over time by *S. obliquus*, demonstrating that the algae efficiently removed Cd^2+^ from the medium, with removal efficiency ranging between 67.6% and 98.6%. Notably, at cadmium concentrations below 0.5 mg L^−1^, the removal rate surged within the first 24 hours before stabilizing, whereas at concentrations above 0.5 mg L^−1^, the trend was reversed.

Figure 5b showed the temporal changes in Cd levels on the cell surface following exposure to varying concentrations of Cd. The data indicated swift adsorption of Cd^2+^ onto the cell surface, with initially high adsorption percentages that subsequently declined. Generally, the adsorption rate increased with higher Cd^2+^ concentrations, peaking at the experiment’s outset with a 10 mg L^−1^ concentration, where it reached 93.2% of the initial concentration.

Concurrently, as shown in Figure 5c, Cd^2+^ was internalized by the cells, with intracellular accumulation rising over time but diminishing at higher Cd^2+^ concentrations. Initially, at 0 h, the accumulation percentage of Cd^2+^ was 15.3% of the initial spiked amount for the lowest concentration group (0.005 mg L^−1^) and less than 1.0% for concentrations above 0.01 mg L^−1^. The accumulation percentage gradually increased and then decreased, reaching its peak at 72 h. The highest accumulation, 83.5%, was observed in the 0.01 mg L^−1^ treatment group.

### 3.6. Composition of Functional Groups

**Figure 6:**
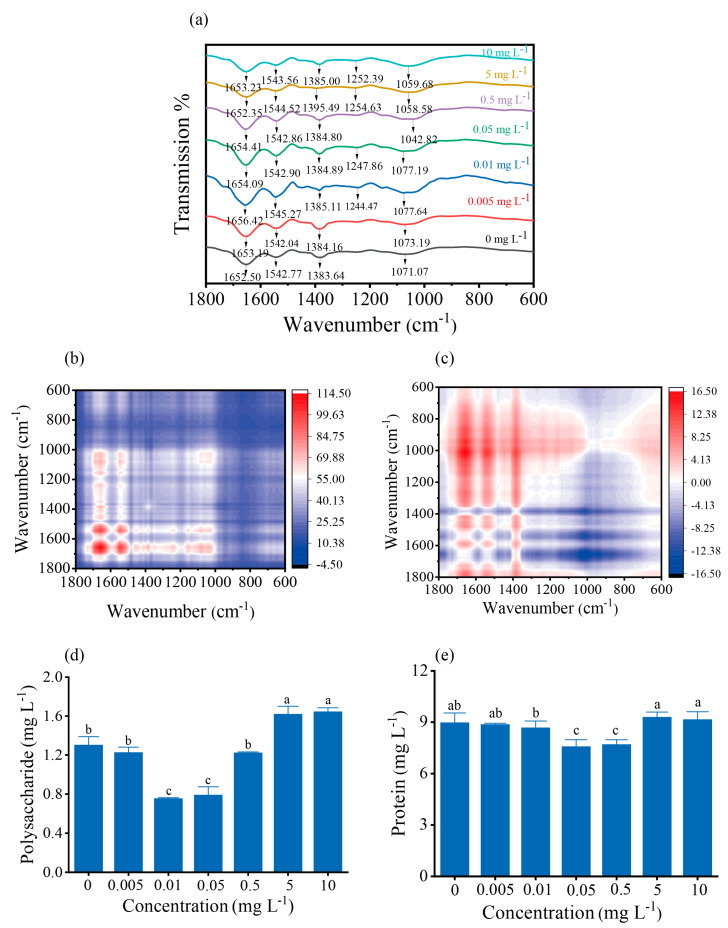
Effects of exposure to different concentrations of Cd^2+^ on the functional groups on the cell surface of *S. obliquus*: (**a**) Fourier infrared spectroscopy, (**b**) synchronous 2D–FTIR–COS map, (**c**) asynchronous 2D–FTIR–COS map, (**d**) polysaccharide content of EPS polysaccharides, (**e**) protein content of EPS. The red and blue dots represent positive and negative correlations, respectively. Higher color intensity indicates a stronger positive or negative correlation. Each data point in the histogram is the mean ± SE of three replicate samples. Different letters above columns represent statistically significant differences, *p* < 0.05.

We also explored the biosorption mechanism on the cell surface using Fourier transform infrared spectroscopy (FTIR). It was found that cell surface polysaccharides and proteins play an important role in cadmium binding. As shown in Figure 6a, the peaks at 1652.50, 1542.77, and 1383.64 cm^−1^ on the algal cell surface were weakened or disappeared, indicating the involvement of proteins and polysaccharides [31] in Cd complexation. 2D−FTIR−COS was employed to distinguish the major functional groups complex with Cd^2+^. The synchronous map (Figure 6b) showed that Cd^2+^ mainly affected the four major auto peaks at 1655, 1542, 1074, and 1053 cm^−1^. The color of two auto peaks of 1655 and 1542 cm^−1^ was darker than that of 1074 and 1053 cm^−1^, suggesting that the band intensities of 1655 cm^−1^ (protein C=O) and 1542 cm^−1^ (protein N−H) changed more significantly under a given Cd^2+^ concentration. The asynchronous map can determine the sequence of spectral intensity change with external perturbation. As shown in Figure 3c, 1655 and 1542 cm^−1^ changed first. In addition, the extracellular proteins were 4 to 8 times more abundant than the exopolysaccharides and both contents gradually increased under the stress of 5 and 10 mg L^−1^ Cd^2+^. (Figure 6d,e). The above results suggest that the carboxyl and amino groups of proteins play an important role in the complexation of Cd^2+^.

## 4. Discussion

This study explored how *S. obliquus* reacts to Cd^2+^ stress, examining morphological alterations, growth suppression, photosynthesis impact, antioxidant mechanisms, and cadmium adsorption and biosorption processes. Findings indicated that while low cadmium levels barely affected the algae, higher concentrations led to notable morphological transformations, decreased growth and photosynthetic rates, and triggered antioxidant defenses. High cadmium removal efficiency was achieved through biosorption and bioaccumulation at lower concentrations (<0.5 mg L^−1^), with biosorption becoming predominant at higher levels (≥0.5 mg L^−1^), facilitated by key roles of cell surface proteins and polysaccharides in cadmium binding. This study underscores *S. obliquus*’s sophisticated response to Cd^2+^ stress, emphasizing its bioremediation capabilities.

Heavy metal contamination of aquatic environments is a serious environmental problem. Cadmium levels in groundwater are as high as 0.005 mg L^−1^ [32]. Furthermore, contaminated systems can reach 1–7 mg L^−1^ [33]. For example, cadmium concentrations in contaminated water bodies in Adamawa State, Nigeria were as high as 1.481 mg L^−1^ [34]. In different types of industrial wastewater, cadmium concentrations ranged from 0.1 to 100 mg L^−1^ [35,36]. Therefore, we explored the bioremediation potential of *S. obliquus* across different cadmium concentrations.

The EC_50_ value indicates the concentration at which cell growth is 50% inhibited and is widely used as a toxicity index to compare the resistance/tolerance of microalgal species in the presence of specific metals. For example, the EC_50_ value of *Desmodesmus pleiomorphus* was 0.058 mg L^−1^ [37]. The EC_50_ values for four green algae, *Ankistrodesmus fusiformis*, *Chlorella ellipsoidea*, *Monoraphidium contortum,* and *Scenedesmus acuminatus* in the contaminated Matanza-Riachuelo River were 0.141 mg L^−1^, 0.429 mg L^−1^, 0.191 mg L^−1^and 0.397 mg L^−1^, respectively [38]. The EC_50_ for *Planothidium lanceolatum* and *Parachlorella kessleri* Bh-2 were found to be 0.25 mg L^−1^ [39] and 0.3 mg L^−1^ [40], respectively. In this study, the EC_50_ of *S. obliquus* was 0.41 ± 0.03 mg L^−1^, which meant that *S. obliquus* has a high tolerance to Cd^2+^ stress.

Toxic stress from cadmium decreases growth and PSII activity in phototrophic organisms [41]. Our findings confirmed that lower concentration (≤0.5 mg L^−1^) of Cd^2+^ had no significant effect on *S. obliquus*, but higher concentrations significantly reduced photosynthetic pigments. The replacement of Mg^2+^ with cadmium in the chlorophyll center disrupts photosynthesis [42,43,44], a mechanism confirmed by studies on cadmium-chlorophyll complexes [45]. At the same time, reactive oxygen species (ROS), a byproduct of chloroplast and mitochondrial metabolism, are produced in large quantities along with altered photosynthetic electron transport activity, indirectly affecting other metabolic activities [46]. Cells respond to this ROS accumulation by activating antioxidant defense mechanisms. Interestingly, low levels of cadmium stress did not induce oxidative stress in cells; however, as cadmium concentration rises, ROS accumulation accelerated, prompting cells to enhance the activity of antioxidant enzymes like SOD (superoxide dismutase) and CAT (catalase) to counteract ROS. Despite this, as cadmium concentration continues to increase, the rate of ROS production within cells escalated, and even with elevated antioxidant enzyme activity, it may not suffice to effectively scavenge the rapidly accumulating ROS. This challenge arises from the diverse nature of ROS, including superoxide anion, hydrogen peroxide, singlet oxygen, and hydroxyl radicals, each requiring specific antioxidant enzymes for neutralization [47]. Because each antioxidant (e.g., vitamin E, SOD, CAT, etc.) is selective for specific types of ROS, it may block one type of ROS but leave another unharmed [48]. In addition, the limited distribution of antioxidant enzymes within cells may hinder their access to ROS–producing sites, particularly within organelles like mitochondria and the chloroplast, which are the primary sites of ROS generation [49]. The total protein content of *S. obliquus* decreased with increasing Cd^2+^ concentration, likely due to the algae reallocating carbon fluxes to synthesize storage molecules such as carbohydrates and lipids to survive under stress. This protein decline may also result from a carbon skeleton shortage due to reduced photosynthesis. The reduced protein content further elucidates why enhanced enzyme activity alone may not be adequate to combat oxidative stress, as some antioxidant enzymes rely on the regeneration of small-molecule antioxidants (e.g., glutathione). The diminished protein content limits the regeneration of these antioxidants, meaning that even with increased enzyme activity, efficient and consistent ROS scavenging may not be achieved.

Morphological changes also confirmed the complex response of *S. obliquus* to Cd^2+^. Under low cadmium stress, *S. obliquus* formed coenobia. With increasing cadmium concentration, the cells gradually crumpled, the plasmalemma wall separated, and the cells converted to a unicellular structure and no longer formed coenobia, which may be because the unicellular morphology is more favorable for nutrient utilization and photocompetition [50]. This may also be because single cells provide more binding sites for the complexation of Cd^2+^ [51]. We also observed the rapid degradation of chloroplasts and mitochondria, the accumulation of electron-dense particles, and the production of starch granules under Cd^2+^ stress. The accumulation of starch granules is usually considered as a protection for cells under stress conditions [40]. Upon exposure to Cd^2+^, accumulating starch granules can serve as a cellular energy store following damage to chloroplasts and mitochondria. Therefore, the accumulation of intracellular starch and high electron density particles may be another effective detoxification method.

Due to the high tolerance of *S. obliquus* to cadmium, we investigated the algae’s capabilities for metal removal, adsorption, and accumulation, finding that *S. obliquus* exhibits high capacities for cadmium removal, adsorption, and accumulation. At initial exposure concentrations of 0.05 mg L^−1^, the cadmium removal efficiency reaches up to 98.6%, and even when the initial exposure concentration is increased to 10 mg L^−1^, the removal efficiency can still reach 67.6%. The study reported that the removal of Cd^2+^ by *Chlorella vulgaris* and *Scenedesmus *sp. at an initial concentration of 1 mg L^−1^ was 8.07% and 5.13%, respectively [52]. *Scenedesmus *sp. IITRIND2, under cultivation conditions with initial concentrations of 5, 10, and 25 mg L^−1^, achieved a Cd removal efficiency of 50–60% within four days [53]. This result suggested that the high removal efficiency of Cd by *S. obliquus* is a good algal resource for the bioremediation of heavy metals.

Biosorption and bioaccumulation are the two main mechanisms reported for metal removal in algae. At the highest initial cadmium concentration (10 mg L^−1^), *S. obliquus* was able to remove about 93.2% (9.32 mg L^−1^) of Cd^2+^ by cell surface adsorption within 24 h, Then, as time extended, the level of biosorption gradually decreased. In contrast to extracellular adsorption, the percentage of intracellular accumulation increased with the treatment time, reaching a peak of 83.5% of the initial concentration of 10 mg L^−1^ at 72 h. This result is higher than that of other algal species; for instance, studies on the removal of cadmium by *Chlorella pyrenoidosa* and *Scenedesmus acutus* found their removal efficiency to be 65.5% and 70.13%, respectively, but bioaccumulation only accounted for 3% and 1.5% of the total removal [54]. This study found that when the Cd concentration was below 0.5 mg L^−1^, *S. obliquus* removed Cd through a combination of adsorption and bioaccumulation. Conversely, cadmium removal was primarily through biosorption when the Cd^2+^ concentration was above 0.5 mg L^−1^.

The biosorption capacity was related to the properties of the EPS [55], with the polysaccharides and proteins in the EPS increased under metal stress, thereby enhancing metal adsorption [56,57]. Studies have shown that the exudates secreted by algae significantly reduce the content of free cadmium, that is, reducing the bioavailability of cadmium, thus affecting the accumulation and toxicity of cadmium in algae and other aquatic organisms [58]. Our study found that the content of proteins in EPS was several times higher than that of polysaccharides and increased under both 5 and 10 mg L^−1^ Cd^2+^ stress, indicating that under cadmium stress, algae attempt to secrete more EPS, including proteins and polysaccharides, to adsorb Cd on the cell surface, preventing metal ions from binding and being transported into the cell, thus protecting the cell from cadmium toxicity. FTIR spectra analysis confirmed that the functional groups such as C=O, C=C, and C–O–C on the cell surface of *S. obliquus* involved in the Cd biosorption and that proteins with C=O and N–H played a dominant role in EPS–Cd complexation. In this way, algae can effectively immobilize Cd^2+^ on the cell surface, reducing their concentration in the water body, as well as their bioavailability, thus playing an important role in the cadmium detoxification process.

## 5. Conclusions

Low concentration of Cd^2+^ (<0.5 mg L^−1^) had no significant effect on *S. obliquus*, as biosorption and bioaccumulation synergistically removed Cd^2+^. Higher concentrations of Cd^2+^ (≥0.5 mg L^−1^) inhibited the growth and photosynthetic electron transport of *S. obliquus*, leading to changes in cell morphology, such as the disappearance of mitochondria and deformation of chloroplasts. *S. obliquus* counters Cd toxicity through antioxidant defense, starch and high–density particle accumulation, and secretion of EPS, which binds Cd^2+^ to surface carboxyl and amino groups.

## Figures and Tables

**Table 1:** Formulae and terms used in the analysis of the O–J–I–P fluorescence induction dynamics curve.

Formulae and Terms	Illustrations
Fo	Minimal record fluorescence intensity
Fm	Maximal recorded fluorescence intensity
$\mathrm{Mo}=4{\;(F}_{300\mu s}-F_{O})\;/\;(\mathrm{Fm}-\mathrm{Fo})$	Approximated initial slope of the fluorescence transient
Sm=(Area ) /(Fm − Fo)	Normalized total complementary area above the O-J-I-P transie (reflecting single-turnover Q_A_ reduction events)
N	The number of times Q_A_ was restored from the time illumination began until Fm arrived.
$\varphi_{\mathrm{Po}}=1-\mathrm{Fo}/\mathrm{Fm}$	Maximum quantum yield for primary photochemistry (at t = 0)
$\psi_{o}=1-\mathrm{Vj}$	The probability that a trapped exciton moves an electron into the electron transport chain beyond Q_A_^−^ (at t = 0)
$\varphi_{E_{o}}=\left(1-\mathrm{Fo}/\mathrm{Fm}\right)\cdot \psi_{o}$	Quantum yield for electron transport (at t = 0)
$\varphi_{D_{o}}=1-\varphi_{P_{o}}$	Quantum yield for dissipated energy (at t = 0)
$\mathrm{ABS}/\mathrm{RC}=\mathrm{Mo}\cdot (1/\mathrm{Vj})\cdot (1/\varphi_{P_{o}})$	Absorption flux per RC
DIo/RC=ABS/RC−TRo/RC	Dissipated energy flux per RC (at t = 0)
TRo/RC=Mo·(1/Vj)	Trapped energy flux per RC (at t = 0)
$\mathrm{ETo}/\mathrm{RC}=\mathrm{Mo}\cdot (1/\mathrm{Vj})\cdot \psi_{o}$	Electron transport flux per RC (at t = 0)
ABS/CSo≈Fo	Absorption flux per CS (at t = 0)
$\mathrm{TRo}/\mathrm{CSo}=\varphi_{p_{o}}\cdot (\mathrm{ABS}/\mathrm{CSo})$	Trapped energy flux per CS (at t = 0)
$\mathrm{EToCSo}=\varphi_{E_{o}}\cdot (\mathrm{ABS}/\mathrm{CSo})$	Electron transport flux per CS (at t = 0)
DIo/CSo=ABS/CSo−TRo/CSo	Dissipated energy flux per CS (at t = 0)
$\mathrm{RC}/\mathrm{CSo}=\varphi_{P_{o}}\cdot (\mathrm{Vj}/\mathrm{Mo})\cdot (\mathrm{ABS}/\mathrm{CSo})$	Density of RCs (Q_A_^−^ reducing PSII reaction centers)
${\mathrm{PI}}_{\mathrm{ABS}}=(\mathrm{RC}/\mathrm{ABS})\cdot \left[\varphi_{P_{o}}/(1-\varphi_{P_{o}})\right]\cdot \left[\psi_{o}/\left(1-\psi_{o}\right)\right]$	Performance index on absorption basis

## Data Availability

Data are contained within the article and Supplementary Materials.

## Supplementary Materials

The following supporting information can be downloaded at: https://www.mdpi.com/article/10.3390/toxics12040262/s1, Table S1: JIP parameter changes of *S. obliquus* treated with different concentrations of Cd^2+^ for 96 h (percentage relative to control); Table S2: *S. obliquus* cells were treated with different concentrations of Cd^2+^ for 96 h and relaxation of the flash-induced fluorescence yield with or without 20 μM DCMU was measured; Figure S1: A simplified Z-scheme of the light reactions of photosynthesis mimicking (Stirbet and Govindjee, 2011); Figure S2: Polyphasic chlorophyll a fluorescence induction kinetics (FI) profile of *S. obliquus* treated with different concentrations of Cd^2+^ for 96 h.
