# Supplementary material for: Cadmium-Induced Physiological Responses, Biosorption and Bioaccumulation in Scenedesmus obliquus
Source: Toxics. 2024 Mar 31;12(4):262. doi: 10.3390/toxics12040262 (PMC11054603; doi:10.3390/toxics12040262)
Supplement: Supplementary file 1 [file toxics-12-00262-s001.zip › toxics-2899043-supplementary.pdf]

Supplementary Material

Cadmium-Induced Physiological Responses, Biosorption and Bioaccumulation in *Scenedesmus obliquus*

Pingping Xu [1,2], Xiaojie Tu [3], Zhengda An [4], Wujuan Mi[1], Dong Wan[1], Yonghong Bi[1] and Gaofei Song [1,*]

[1] State Key Laboratory of Freshwater Ecology and Biotechnology, Institute of Hydrobiology, Chinese Academy of Sciences, Wuhan 430072, China; xupingping@ihb.ac.cn (P.X.); miwj@ihb.ac.cn (W.M.); wandong@inb.ac.cn (D.W.); biyh@ihb.ac.cn (Y.B.)
[2] University of Chinese Academy of Sciences, Beijing 100049, China
[3] Geophysical Exploration Brigade of Hubei Geological Bureau, Wuhan, 430056, China; tuxj@ihb.ac.cn
[4] College of Life Science, Wuhan University, Wuhan 430072, China; 2020300002057@whu.edu.cn
* Correspondence: song@ihb.ac.cn

Table S1. JIP parameter changes of *S. obliquus* treated with different concentrations of Cd^{2+} at 96 h (percentage relative to control)

name	0 mg L^{-1}	0.005 mg L^{-1}	0.01 mg L^{-1}	0.05 mg L^{-1}	0.5 mg L^{-1}	5 mg L^{-1}	10 mg L^{-1}
Fo	100.00%	136.73%	98.62%	98.75%	110.58%	84.79%	52.69%
Fm	100.00%	92.47%	99.71%	99.84%	95.17%	41.90%	20.03%
V_J	100.00%	144.07%	99.01%	97.43%	113.24%	264.43%	303.36%
V_I	100.00%	107.21%	100.30%	96.43%	97.68%	140.98%	146.81%
Mo	100.00%	167.30%	98.64%	97.55%	120.16%	349.86%	442.51%
Sm	100.00%	158.56%	103.98%	100.76%	114.36%	305.86%	487.08%
N	100.00%	183.77%	103.76%	100.62%	121.38%	404.59%	715.58%
φ_{Po}	100.00%	85.51%	100.30%	100.30%	95.05%	68.85%	48.94%
ψ_o	100.00%	91.06%	100.20%	100.52%	97.31%	66.64%	58.74%
φ_{Eo}	100.00%	77.91%	100.57%	100.89%	92.58%	45.90%	28.98%
φ_{Do}	100.00%	148.06%	98.99%	98.99%	116.40%	203.31%	269.35%
PI_{ABS}	100.00%	17.78%	103.20%	105.06%	63.57%	4.54%	1.35%
ABS/RC	100.00%	136.60%	100.09%	100.23%	112.38%	193.32%	307.90%
TRo/RC	100.00%	115.93%	99.72%	99.82%	106.06%	132.05%	145.32%
ETo/RC	100.00%	105.58%	99.89%	100.39%	103.26%	87.96%	85.08%
DIo/RC	100.00%	200.61%	98.48%	98.48%	129.98%	390.72%	837.75%

Table S2. *S. obliquus* cells were treated with different concentrations of Cd^{2+} at 96 h and relaxation of the flash-induced fluorescence yield with or without 20 μM DCMU was measured. The curves were analyzed in terms of three exponential components (fast, middle, and slow phases).

Concentration mg L^{-1}	Fast phase T_1(μs)/A_1(%)	Middle phase T_2(ms)/A_2(%)	Slow phase T_3(s)/A_3(%)	A_0(%)
Without DCMU				
0	22.40±3.23/23.74%	0.35±0.03/40.57%	0.10±0.02/23.72%	11.97%
0.005	25.67±3.50/25.65%	0.37±0.03/39.38%	0.10±0.02/22.03%	12.95%
0.01	27.87±3.80/26.75%	0.38±0.04/39.17%	0.10±0.02/21.56%	12.52%
0.05	21.93±3.05/24.81%	0.40±0.03/39.39%	0.09±0.02/22.27%	13.54%
0.5	17.01±2.78/24.96%	0.37±0.03/39.94%	0.06±0.01/22.81%	12.29%
5	\	\	\	\
10	\	\	\	\
With DCMU				
0			0.46±0.03/60.3%	39.7%
0.005			0.46±0.03/60.2%	39.8%
0.01			0.46±0.03/60.02%	39.8%
0.05			0.50±0.03/59.7%	40.3%
0.5			0.49±0.03/59.9%	40.1%
5			0.51±0.05/58.8%	41.2%
10			0.77±0.19/59.9%	40.1%

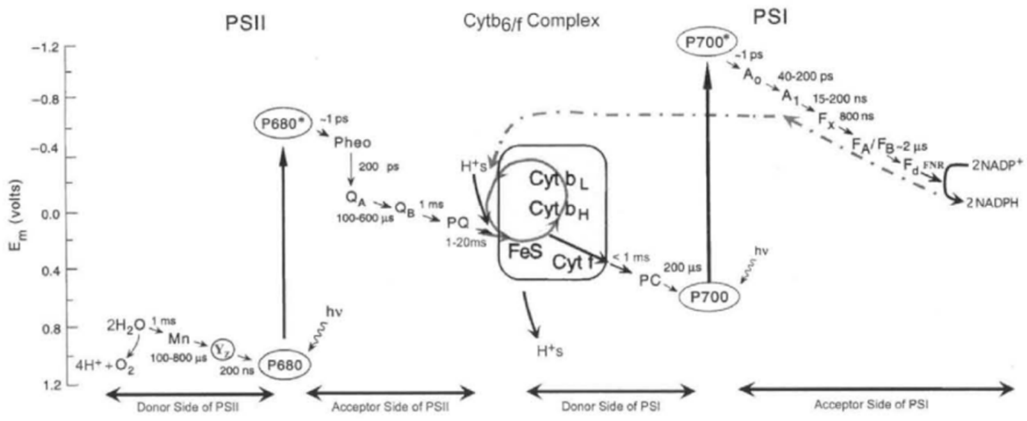

Figure S1. A simplified Z-scheme of the light reactions of photosynthesis mimicking (Stirbet and Govindjee, 2011).

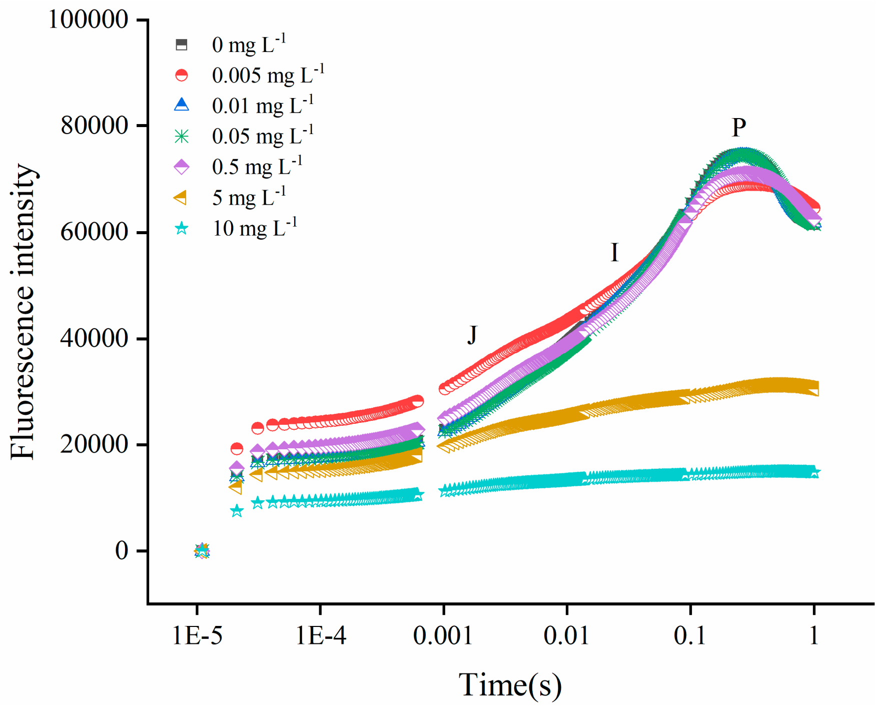

Figure S2. Polyphasic chlorophyll a fluorescence induction kinetics (FI) profile of *S. obliquus* treated with different concentrations of Cd^{2+} for 96 h.

References

1. Stirbet, A., and Govindjee, 2011. On the relation between the Kautsky effect (chlorophyll a fluorescence induction) and Photosystem II: Basics and applications of the OJIP fluorescence transient. Journal of Photochemistry and Photobiology B: Biology 104, 236-257.
